# Supplementary material for: Effects of lumbar disc injury and nociception on trunk motor control during rat locomotion
Source: Exp Brain Res. 2026 Jul 4;244(8):149. doi: 10.1007/s00221-026-07350-6 (PMC13332984; doi:10.1007/s00221-026-07350-6)
Supplement: Supplementary file 1 — Supplementary Material 1 [file 221_2026_7350_MOESM1_ESM.docx]

| **a** | 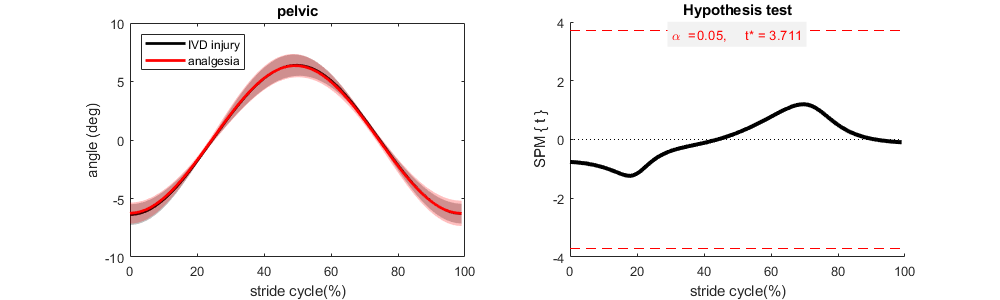 |
| --- | --- |
| **b** | 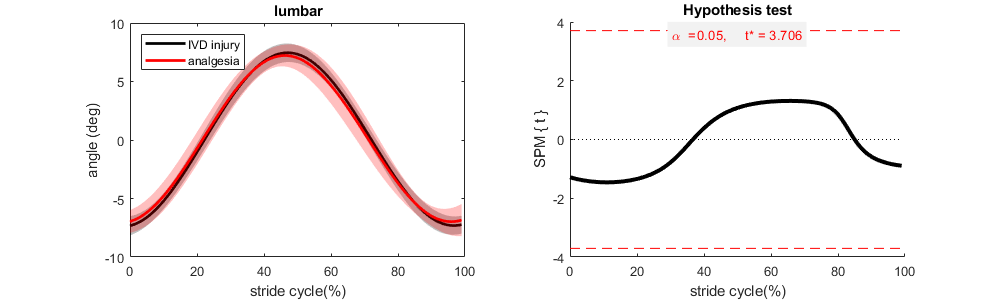 |
| **c** | 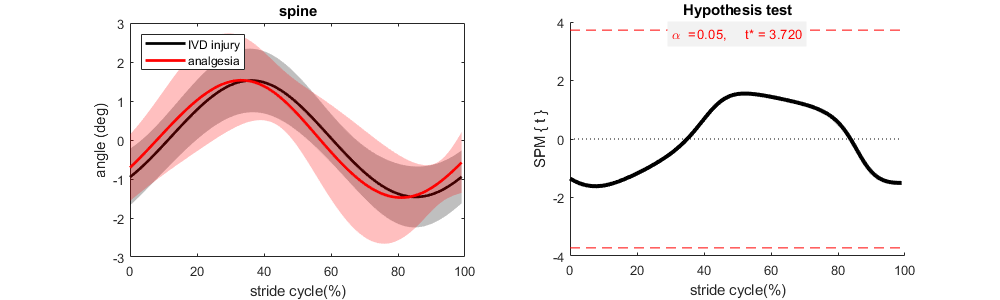 |
| **Fig.S1.** SPM analysis of the segmental angle data during locomotion for IVD injury and analgesia (carprofen injection) conditions. **(a)** pelvic, **(b)** lumbar, **(c)** spine. Joint angle data were averaged cross 10 rats and normalized to stride cycle duration. Shaded area represents mean±1SD. Treadmill speed was at 0.5m/s at all conditions. IVD, intervertebral disc injury. | |

| **a** | 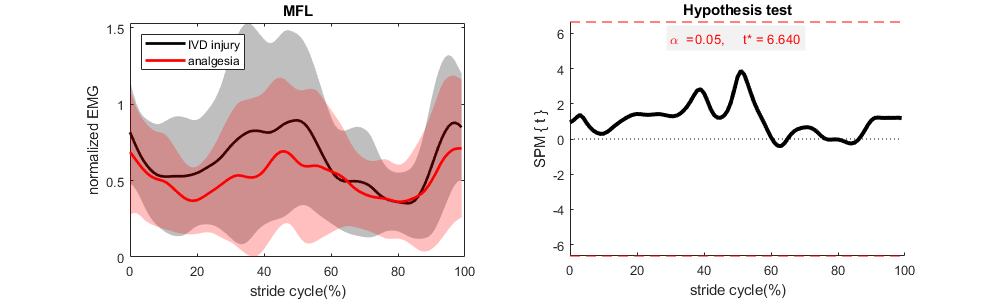 |
| --- | --- |
| **b** | 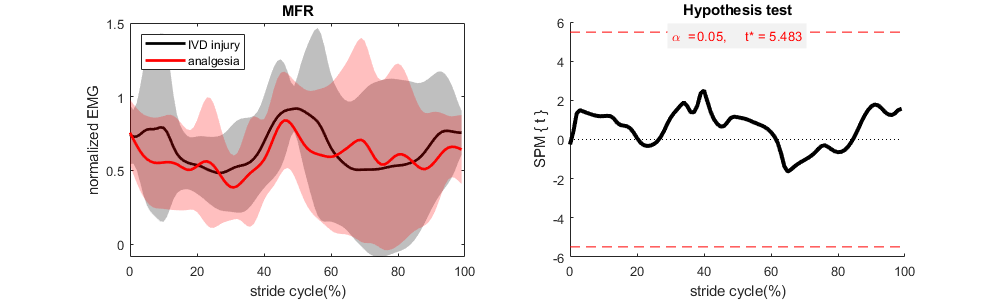 |
| **c** | 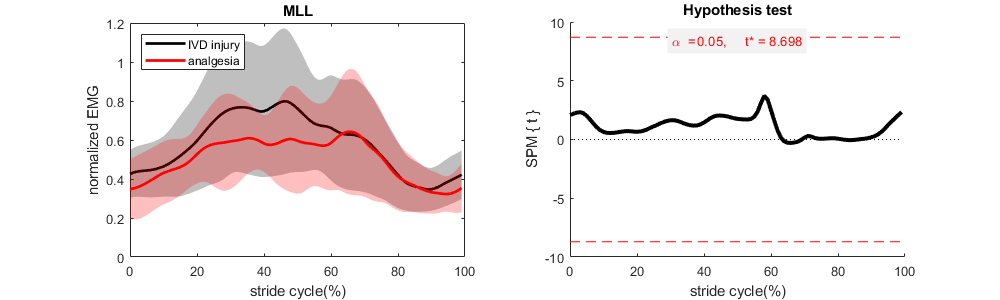 |
| **d** | 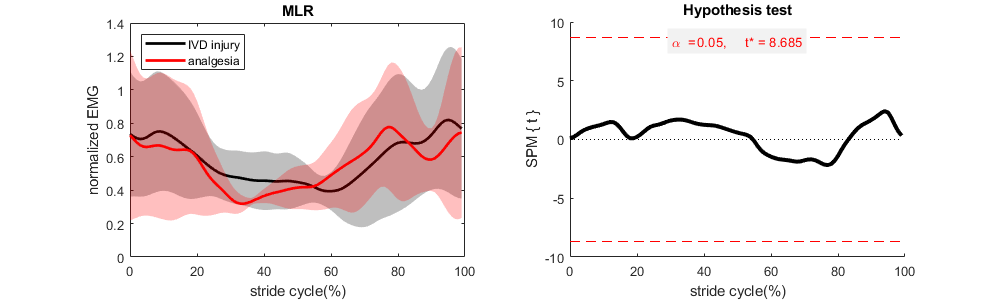 |
| **Fig.S2.** SPM analysis of the filtered rectified EMG envelope of back muscles during locomotion for IVD injury and analgesia (carprofen injection) conditions. **(a)** MFL (multifidus muscle left, n=6), **(b)** MFR (multifidus muscle right, n=8), **(c)** MLL (longissimus muscle left, n=5), **(d)** MLR (longissimus muscle right, n=5). EMG data were normalized to the stride cycle duration and peak amplitude measured during baseline. IVD, intervertebral disc injury. | |

| **a** | 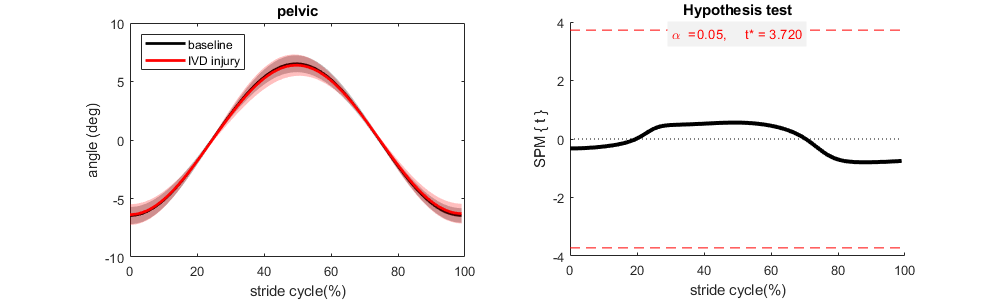 |
| --- | --- |
| **b** | 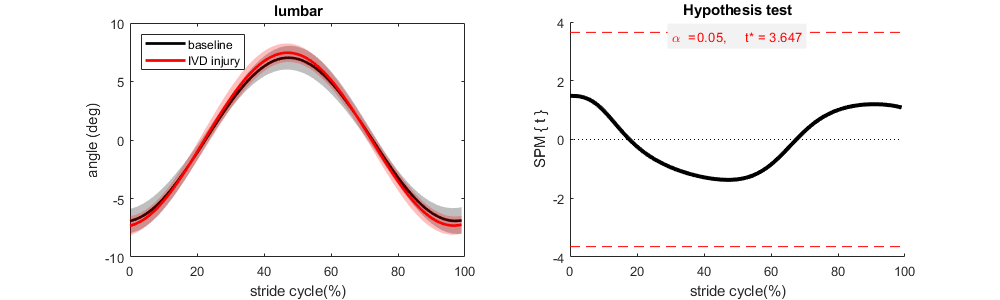 |
| **c** | 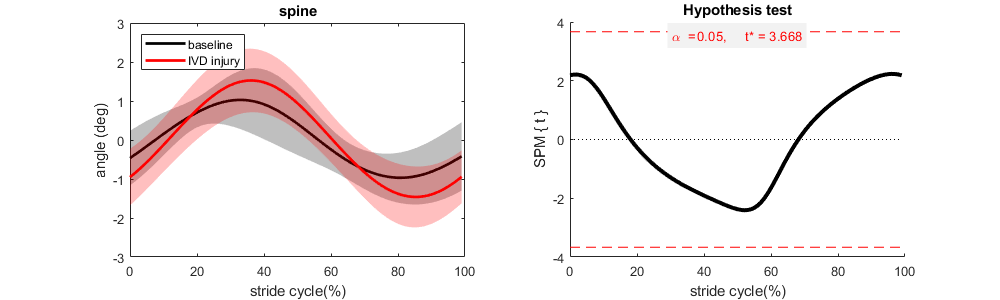 |
| **Fig.S3.** SPM analysis of the segmental angle data during locomotion for baseline and IVD injury conditions. **(a)** pelvic, **(b)** lumbar, **(c)** spine. Joint angle data were averaged cross 10 rats and normalized to stride cycle duration. Shaded area represents mean±1SD. Treadmill speed was at 0.5m/s at all conditions. IVD, intervertebral disc injury. | |

| **a** | 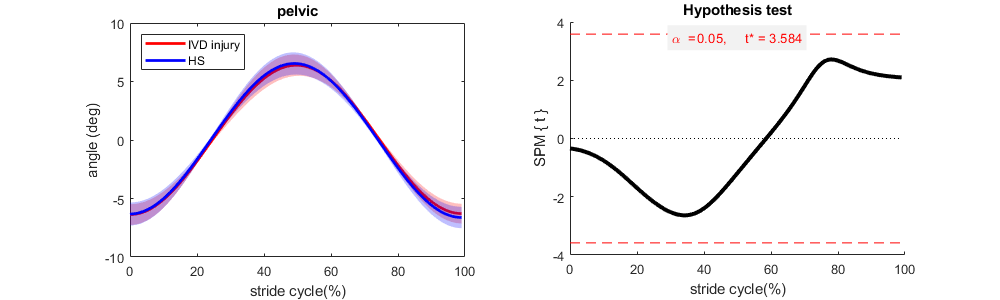 |
| --- | --- |
| **b** | 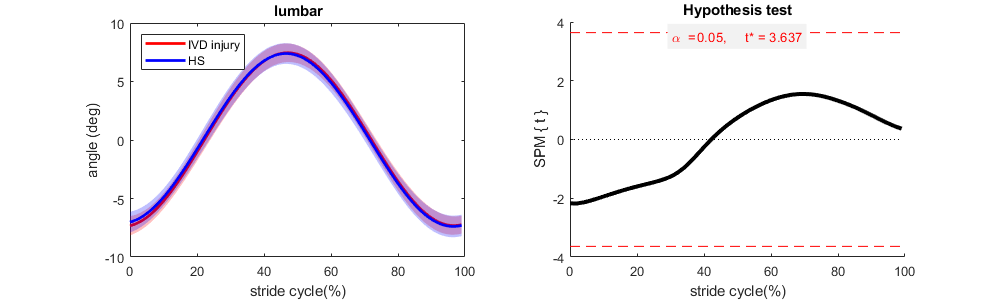 |
| **c** | 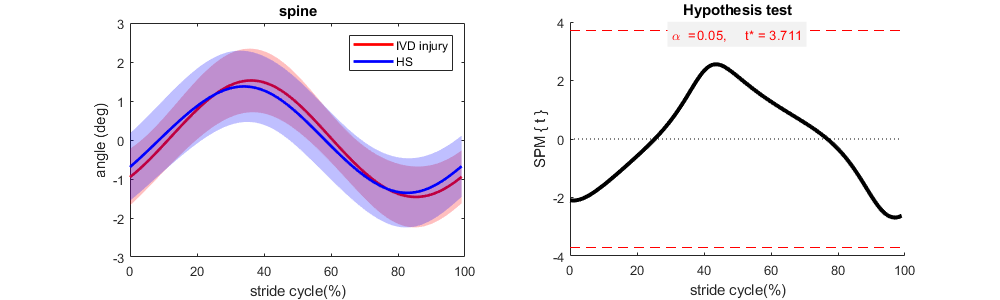 |
| **Fig.S4.** SPM analysis of the segmental angle data during locomotion for IVD injury and IVD injury + hypertonic saline injection (HS) conditions. **(a)** pelvic, **(b)** lumbar, **(c)** spine. Joint angle data were averaged cross 10 rats and normalized to stride cycle duration. Shaded area represents mean±1SD. Treadmill speed was at 0.5m/s at all conditions. IVD, intervertebral disc injury. | |

| **a** | 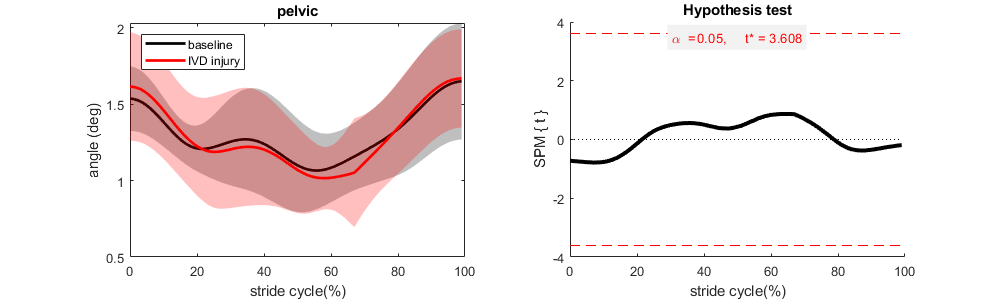 |
| --- | --- |
| **b** | 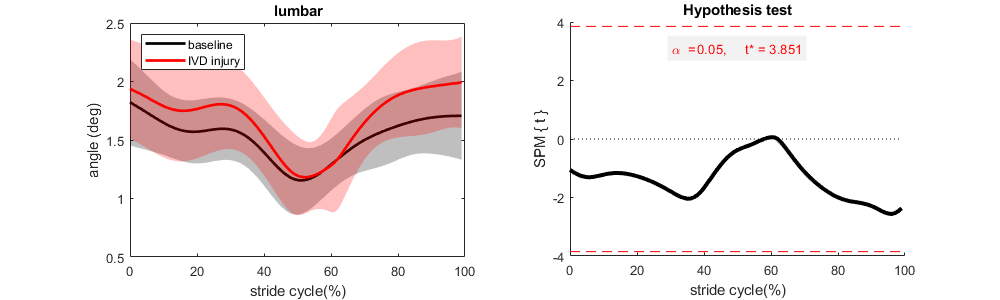 |
| **c** | 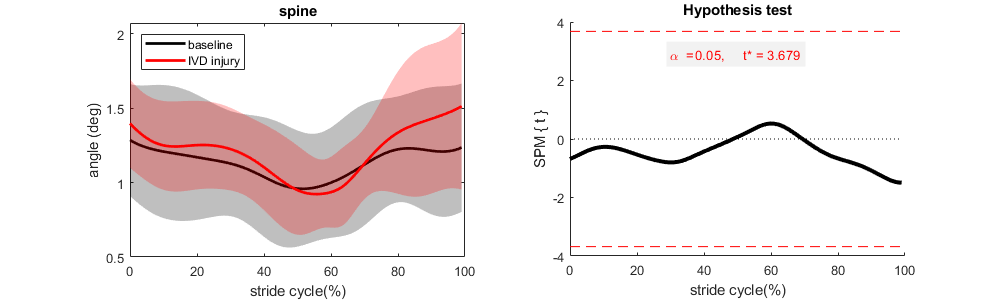 |
| **Fig.S5.** SPM analysis of the segmental angle variability during locomotion for baseline and IVD injury conditions. **(a)** pelvic, **(b)** lumbar, **(c)** spine. Joint angle variability data were averaged cross 10 rats and normalized to stride cycle duration. Shaded area represents mean±1SD. Treadmill speed was at 0.5m/s at all conditions. IVD, intervertebral disc injury. | |

| **a** | 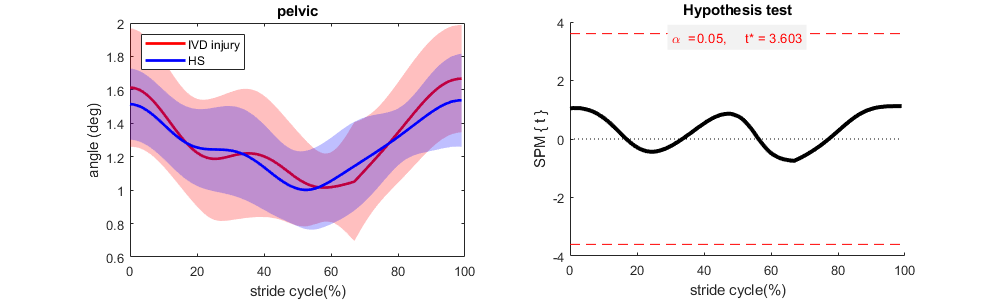 |
| --- | --- |
| **b** | 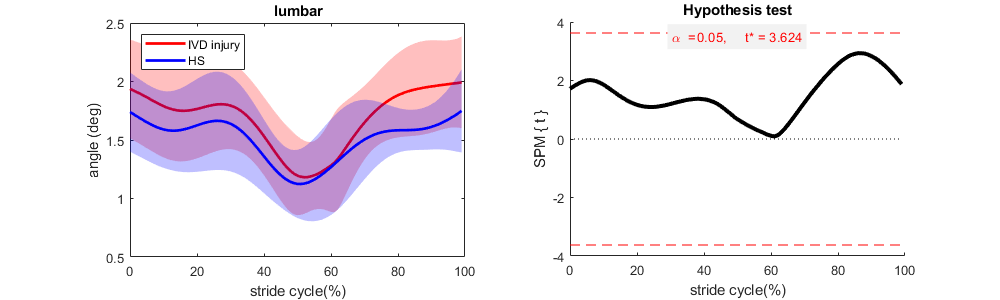 |
| **c** | 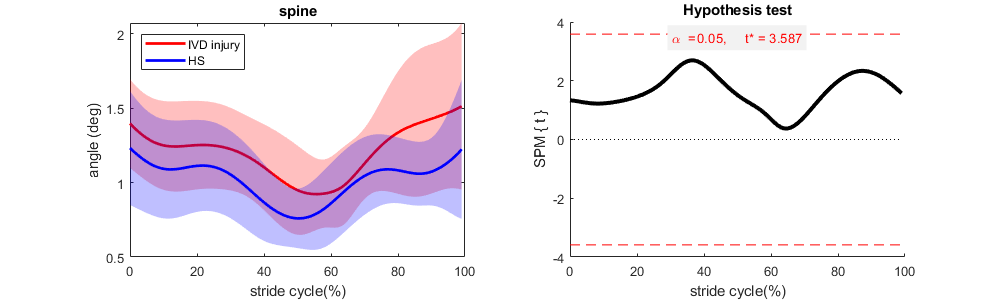 |
| **Fig.S6.** SPM analysis of the segmental angle variability during locomotion for IVD injury and IVD injury + hypertonic saline injection (HS) conditions. **(a)** pelvic, **(b)** lumbar, **(c)** spine. Joint angle variability data were averaged cross 10 rats and normalized to stride cycle duration. Shaded area represents mean±1SD. Treadmill speed was at 0.5m/s at all conditions. IVD, intervertebral disc injury. | |

| **a** | 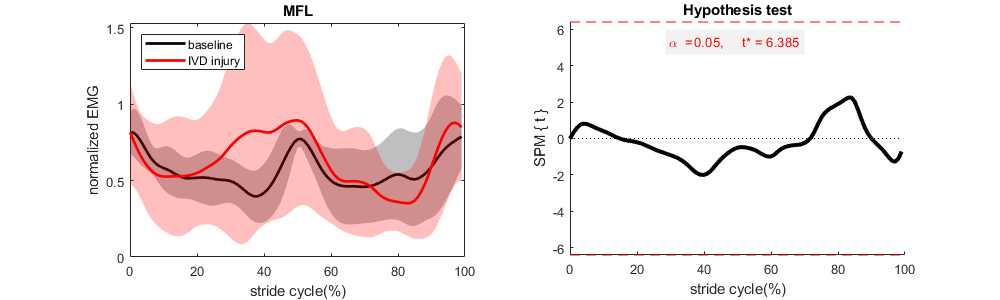 |
| --- | --- |
| **b** | 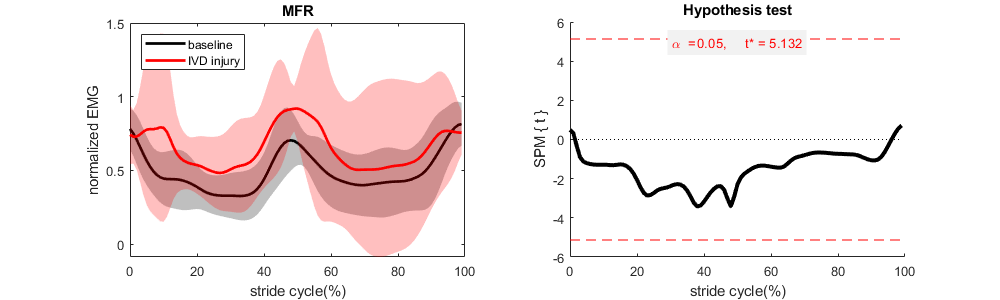 |
| **c** | 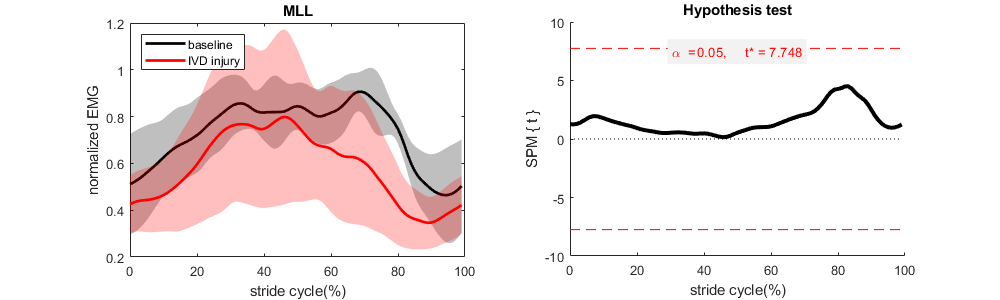 |
| **d** | 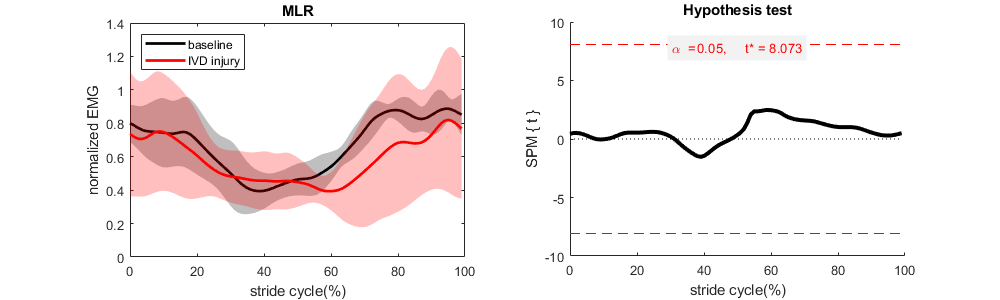 |
| **Fig.S7.** SPM analysis of the filtered rectified EMG envelope of back muscles during locomotion for baseline and IVD injury conditions. **(a)** MFL (multifidus muscle left, n=6), **(b)** MFR (multifidus muscle right, n=8), **(c)** MLL (longissimus muscle left, n=5), **(d)** MLR (longissimus muscle right, n=5). EMG data were normalized to the stride cycle duration and peak amplitude measured during baseline. IVD, intervertebral disc injury. | |

| **a** | 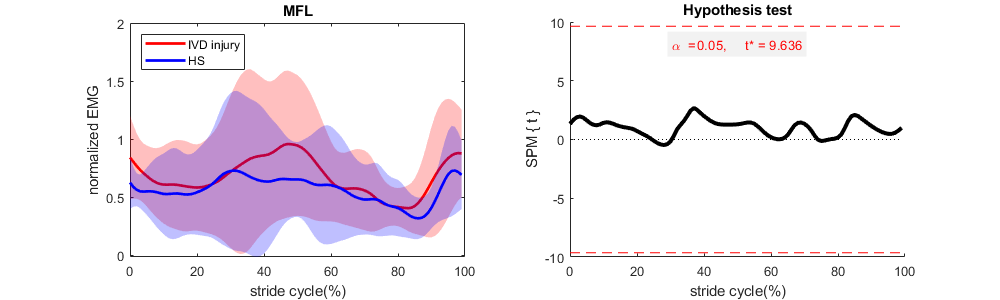 |
| --- | --- |
| **b** | 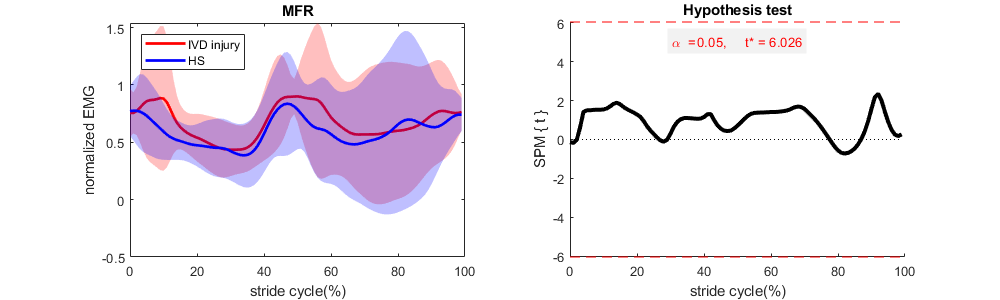 |
| **c** | 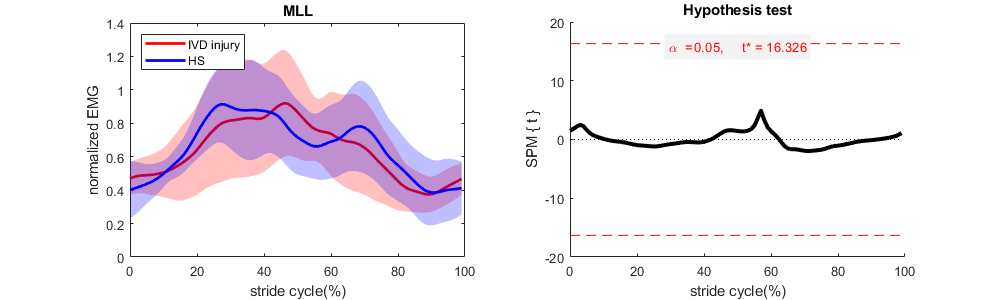 |
| **d** | 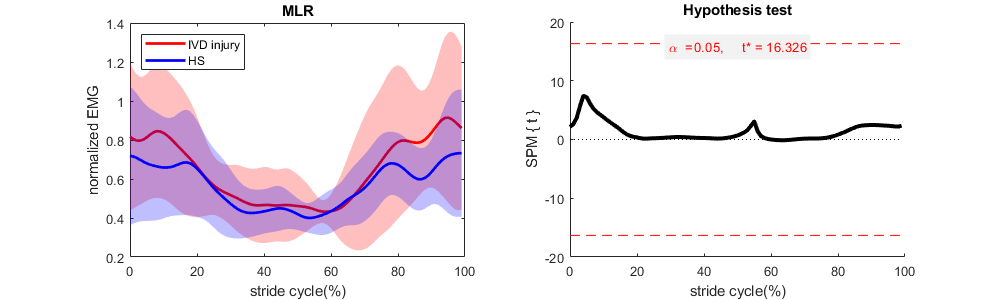 |
| **Fig.S8.** SPM analysis of the filtered rectified EMG envelope of back muscles during locomotion for IVD injury and IVD injury + hypertonic saline injection (HS) conditions. **(a)** MFL (multifidus muscle left, n=5), **(b)** MFR (multifidus muscle right, n=7), **(c)** MLL (longissimus muscle left, n=4), **(d)** MLR (longissimus muscle right, n=4). EMG data were normalized to the stride cycle duration and peak amplitude measured during baseline. IVD, intervertebral disc injury. | |

| **a** | 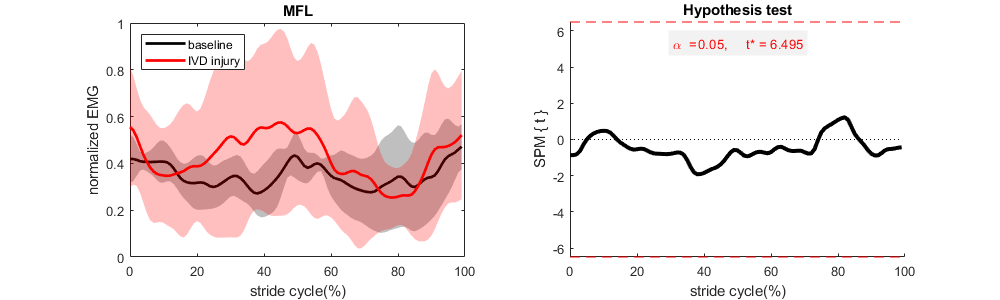 |
| --- | --- |
| **b** | 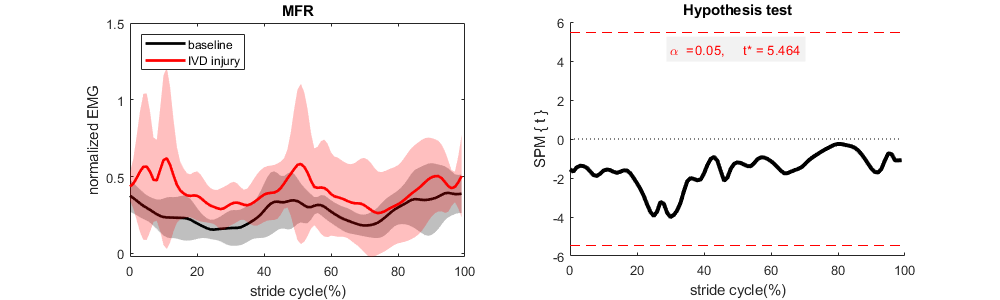 |
| **c** | 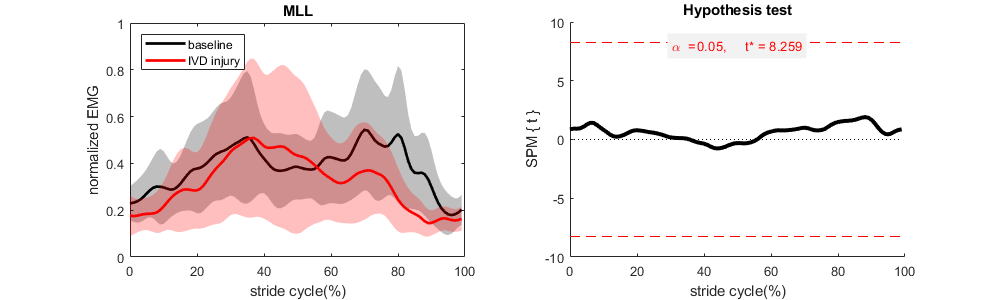 |
| **d** | 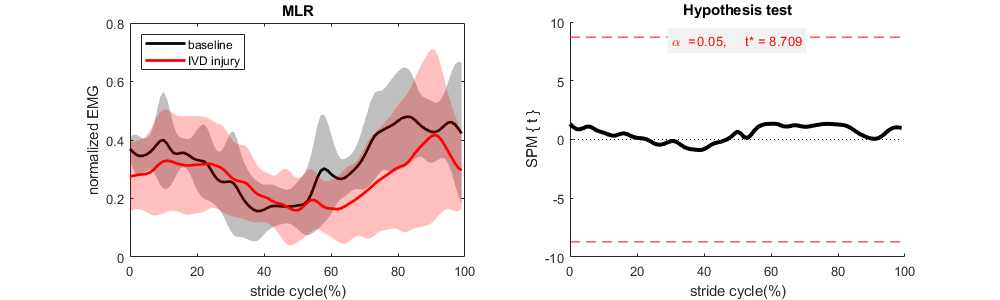 |
| **Fig.S9.** SPM analysis of the EMG variability of back muscles during locomotion for baseline and IVD injury conditions. **(a)** MFL (multifidus muscle left, n=6), **(b)** MFR (multifidus muscle right, n=8), **(c)** MLL (longissimus muscle left, n=5), **(d)** MLR (longissimus muscle right, n=5). EMG data were normalized to the stride cycle duration and peak amplitude measured during baseline. IVD, intervertebral disc injury. | |

| **a** | 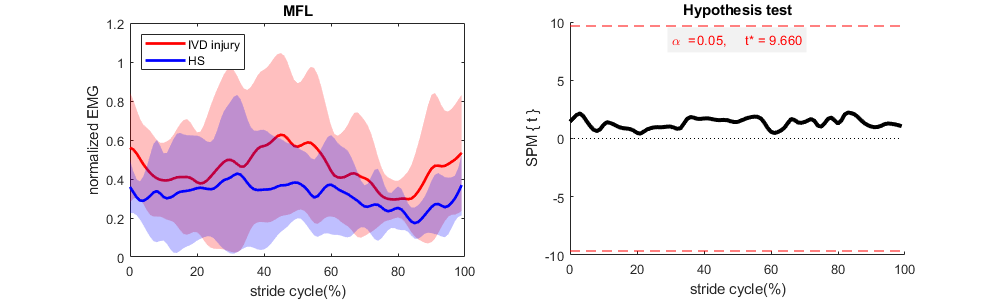 |
| --- | --- |
| **b** | 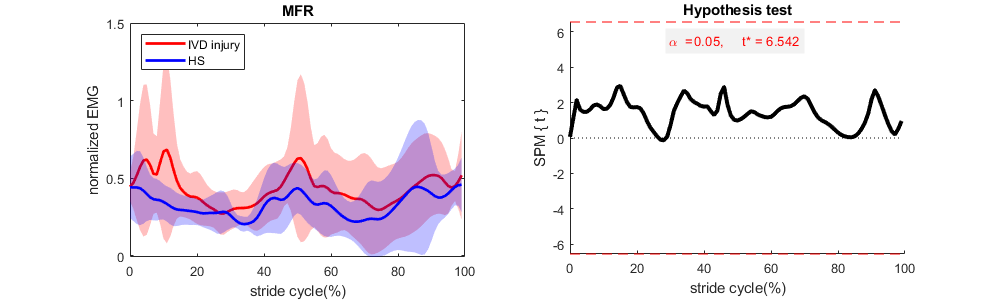 |
| **c** | 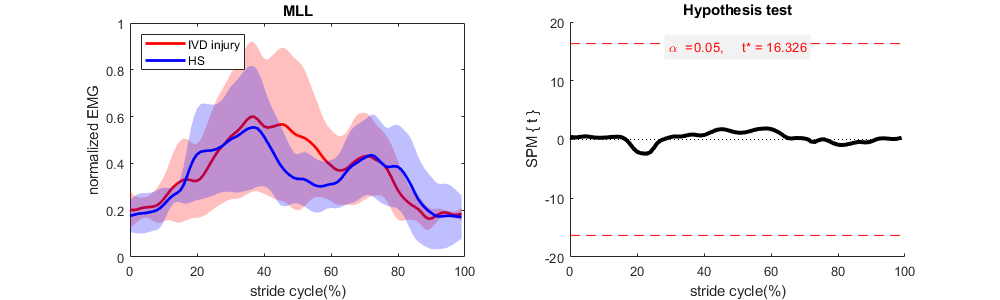 |
| **d** | 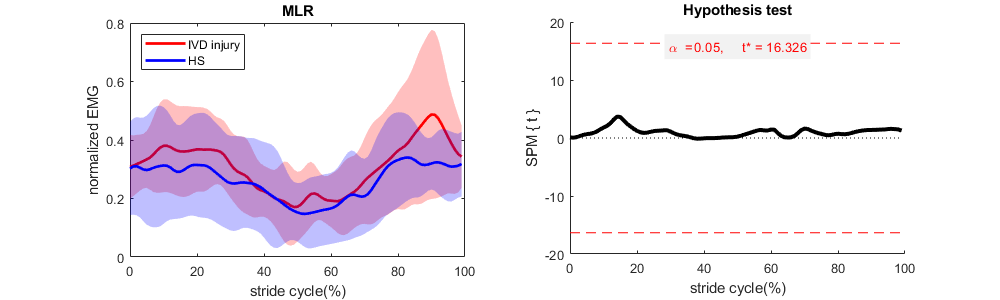 |
| **Fig.S10.** SPM analysis of the EMG variability of back muscles during locomotion for IVD injury and IVD injury + hypertonic saline injection (HS) conditions. **(a)** MFL (multifidus muscle left, n=5), **(b)** MFR (multifidus muscle right, n=7), **(c)** MLL (longissimus muscle left, n=4), **(d)** MLR (longissimus muscle right, n=4). EMG data were normalized to the stride cycle duration and peak amplitude measured during baseline. IVD, intervertebral disc injury. | |
